# Using a Virtual Environment to Deliver Evidence-Based Interventions: The Facilitator's Experience

**DOI:** 10.2196/games.4293

**Published:** 2015-07-21

**Authors:** Michelle Aebersold, Antonia Villarruel, Dana Tschannen, Angel Valladares, Joseph Yaksich, Emily Yeagley, Armani Hawes

**Affiliations:** ^1^ University of Michigan School of Nursing Ann Arbor, MI United States; ^2^ University of Pennsylvania Philadelphia, PA United States; ^3^ University of Michigan Ann Arbor, MI United States

**Keywords:** Second Life, multi-user virtual environments, evidence-based interventions, community-based organizations

## Abstract

**Background:**

Evidence-based interventions (EBIs) have the potential to maximize positive impact on communities. However, despite the quantity and quality of EBIs for prevention, the need for formalized training and associated training-related expenses, such as travel costs, program materials, and input of personnel hours, pose implementation challenges for many community-based organizations. In this study, the community of inquiry (CoI) framework was used to develop the virtual learning environment to support the adaptation of the ¡Cuídate! (Take Care of Yourself!) Training of Facilitators curriculum (an EBI) to train facilitators from community-based organizations.

**Objective:**

The purpose of this study was to examine the feasibility of adapting a traditional face-to-face facilitator training program for ¡Cuídate!, a sexual risk reduction EBI for Latino youth, for use in a multi-user virtual environment (MUVE). Additionally, two aims of the study were explored: the acceptability of the facilitator training and the level of the facilitators’ knowledge and self-efficacy to implement the training.

**Methods:**

A total of 35 facilitators were trained in the virtual environment. We evaluated the facilitators' experience in the virtual training environment and determined if the learning environment was acceptable and supported the acquisition of learning outcomes. To this end, the facilitators were surveyed using a modified community of inquiry survey, with questions specific to the Second Life environment and an open-ended questionnaire. In addition, a comparison to face-to-face training was conducted using survey methods.

**Results:**

Results of the community of inquiry survey demonstrated a subscale mean of 23.11 (SD 4.12) out of a possible 30 on social presence, a subscale mean of 8.74 (SD 1.01) out of a possible 10 on teaching presence, and a subscale mean of 16.69 (SD 1.97) out of a possible 20 on cognitive presence. The comparison to face-to-face training showed no significant differences in participants' ability to respond to challenging or sensitive questions (*P*=.50) or their ability to help participants recognize how Latino culture supports safer sex (*P*=.32). There was a significant difference in their knowledge of core elements and modules (*P*<.001). A total of 74% (26/35) of the Second Life participants did agree/strongly agree that they had the skills to deliver the ¡Cuídate! program.

**Conclusions:**

The results showed that participants found the Second Life environment to be acceptable to the learners and supported an experience in which learners were able to acquire the knowledge and skills needed to deliver the curriculum.

## Introduction

### Overview

Imagine a place where you can attend a fully interactive training session with people in different settings from all areas of the country without having to leave your home or office. You could learn about topics important to your work so you could help others in your community. That place is a virtual environment, a computer-generated three-dimensional representation of a space in which users can interact. They can take advantage of current Web 2.0 technologies, which are technologies focused on user-generated content, to deliver accessible and interactive training for communities and organizations. Training needs of community-based organizations (CBOs) and others can range from information sessions to more intensive training sessions to conduct evidence-based interventions (EBIs).

Evidence-based interventions are programs that have undergone rigorous outcome evaluation and have the potential to maximize positive impact on communities [1]. However, despite the quantity and quality of EBIs for health promotion and disease prevention, the need for formalized training and associated training-related expenses, such as travel costs, program materials, and input of personnel hours, pose implementation challenges for CBOs [2,3].

To increase access to EBI training, cost-effective training methods, such as Web-based training platforms, are needed. Accordingly, advances in technology have resulted in the development of multi-user virtual environments (MUVEs) as platforms for social interaction. The creation of a sense of presence among users [4] has many benefits over less dynamic forms of traditional Web-based trainings such as webinars or asynchronous podcasts. While the use of MUVEs is commonplace among gamers and the technologically savvy, the use of MUVEs among community providers is not widespread. Little is known about the acceptance of MUVEs in community settings and agencies whose staff vary in computer experience and in familiarity and comfort level with virtual training environments.

The purpose of this study was to examine the feasibility of adapting a traditional face-to-face facilitator training program for *¡Cuídate!* (Take Care of Yourself!), a sexual risk reduction EBI for Latino youth [5], for use in a MUVE. Facilitators are individuals who are trained in the delivery of the EBI who then deliver the intervention to youth in their communities. In this study, the community of inquiry (CoI) framework [6] was used to develop the virtual learning environment to support the adaptation of the *¡Cuídate!* face-to-face curriculum. The constructs of the CoI framework (ie, social, teaching, and cognitive presence) were then used as a basis to evaluate if the facilitator learning experience in the 2.5-day MUVE training program was acceptable to the learners and supported the acquisition of learning outcomes. Further comparison was conducted with those facilitators trained in the virtual environment and those trained in face-to-face formats. This, in part, addressed two aims of the study: to examine the acceptability of the facilitator training program and to examine the level of the facilitators’ knowledge and self-efficacy to implement the training.

### Virtual Learning Environment

The CoI [6] is one of the most common frameworks for assessing individual acceptance and comfort with online-learning environments. The model is comprised of three constructs that are core elements of a collaborative constructivist learning environment: (1) cognitive presence—the ability of learners to construct meaning through reflection and discourse, (2) social presence—the ability of participants to feel connected to each other in the absence of face-to-face contact, and (3) teaching presence—the design, facilitation, and direction of processes needed to support learning [7]. Burgess used the CoI survey to determine the extent of social, cognitive, and teaching presence among graduate level technology students in class activities held in Second Life (SL) [8]. These constructs have been positively associated with learning in an online environment. For example, a sense of community has been shown to have a positive relationship with perceived cognitive learning [9] and all three CoI constructs were predictive of perceived learning in online Master of Business Administration (MBA) courses [10]. In another study, Liu and colleagues found social presence was a significant predictor of course retention among students enrolled in community college [11].

The CoI framework and Second Life were purposely chosen to guide the adaption of the *¡Cuídate!* training program because it requires the application of skills and reflection on the work of self and others (cognitive presence), high interaction and social connection among participants (social presence), and real-time feedback from facilitators and peers (teaching presence). These elements are lacking in a traditional online Web-based training environment.

### Second Life

Second Life was developed by Linden Lab and is considered one of the most mature and widely used platforms in use, specifically in health care. Through the creation and use of a modifiable avatar (ie, an online, graphical representation of the user), individuals in SL are able to interact with people and objects with the ability to exhibit social cues through realistic gestures [12].

Second Life has been used in a variety of interventions in health education for both practitioners and patients. Second Life is also not new to the area of sexual health; the University of Plymouth Sexual Health Sim was developed in the United Kingdom as a place to provide sexual health education as well as private one-on-one counseling [13]. Studies using SL for practitioner training have found positive results. For example, one study used SL for motivational interviewing (MI) training among physicians [14]. This training included interactive sessions in SL using role play with standardized patients to practice MI skills and was found to have a high degree of user acceptability. Participants (n=13) rated the acceptability of the various components of the course on a range from 4.1 to 4.7 on a 5-point Likert scale. Proficiency scores in MI also improved, with statistically significant improvement seen in four out of five component skills. In a study with paramedic students, which compared paper-based and SL case scenarios for problem-based learning, participants reported a more authentic and collaborative experience in SL [15]. In addition, 100% of participants surveyed agreed/strongly agreed that SL is a relevant resource for field/clinical work preparation. Similarly, Schwaab and colleagues [16] reported that emergency medicine residents (n=27) participating in mock SL, oral examination case scenarios experienced a high degree of comfort (100%) and realism (92.6%). A majority indicated that SL was easy to navigate (96.3%) and easy to log in to (92.6%), and preferred the SL oral examinations over the traditional format (66.6%). A recently published systematic review of the use of virtual worlds in health care [17] found 11 studies published in the area of professional education, including using virtual worlds for medical education for diabetes [18], delivering bad news to patients [19], and improving patient safety [20].

Given the success of SL in training health care providers and the capacity to create a collaborative and realistic experience for learners, SL was deemed a useful environment to increase dissemination of EBIs among communities. This feasibility study could provide valuable information on the viability of using a MUVE such as SL and on creating a framework for others to use in designing training programs.

## Methods

### Overview

This was a descriptive comparative study to evaluate the feasibility and acceptability of the SL environment, and to compare SL training to face-to-face training. The study protocol was reviewed by the Institutional Review Board at the University of Michigan and was deemed exempt and not regulated.

To examine feasibility and acceptability, data were obtained from the participants (ie, facilitators) who participated in the SL ¡*Cuídate!* Training of Facilitators. For comparison with the face-to-face training process, evaluation data were obtained from the Centers for Disease Control and Prevention (CDC) through Danya International, Inc (personal communication, Danya International, Inc, 2014).

### Training in Second Life

The *¡Cuídate!* Training of Facilitators Manual [21] was used to adapt the 2.5-day, face-to-face training program into a combination of self-paced, prelearning podcasts and live virtual sessions in SL [22]. The *¡Cuídate!* training program was previously only offered as a face-to-face training program; a trainer conducted the 2.5-day session to teach the facilitators (ie, the study participants) how to deliver the curriculum to Latino youths in their communities. The facilitators were asked to review the *¡Cuídate!* curriculum and all the associated activities prior to attending the virtual training sessions. The six modules of the curriculum included the following: (1) Introduction/Overview, (2) Building Knowledge about Pregnancy, Sexually Transmitted Diseases (STDs), HIV, (3) Understanding Vulnerability to Pregnancy, STDs, HIV, (4) Attitudes and Beliefs about Pregnancy, STDs, HIV, (5) Building Condom Use Skills, and (6) Building Negotiation and Refusal Skills. The facilitators received a hard copy of the entire curriculum approximately two weeks before their SL session by Select Media. Each facilitator was assigned to facilitate (ie, role play) several of the activities in the SL *¡Cuídate!* training sessions. Two master trainers, or expert *¡Cuídate!* trainers, conducted the virtual training sessions and previously conducted face-to-face training sessions. These master trainers facilitated the entire training session in SL in English, similar to the face-to-face training sessions.

The training was designed to allow facilitators to deliver and practice facilitating activities in the curriculum, and to receive feedback from their peers and *¡Cuídate!* master trainers similar to what would be experienced in a face-to-face *¡Cuídate!* training session. Specific curricular activities were selected to utilize the capabilities of the interactive virtual environment. Unlike face-to-face training sessions, not all of the activities in every module were completed in the virtual training sessions. Those activities that overlapped in structure and format were minimized in order to demonstrate activities that might be challenging to conduct in real life. Throughout the training session, facilitators were encouraged to interact with each other and the *¡Cuídate!* master trainers by providing feedback to one another and acting as participants (ie, adolescents) during the role play sessions (ie, teach backs). Facilitators were oriented to the SL environment prior to the training sessions and processes; technical support in SL was provided before and during training to minimize any technical issues that might arise during training [23].

### Sample

**Figure 1:**
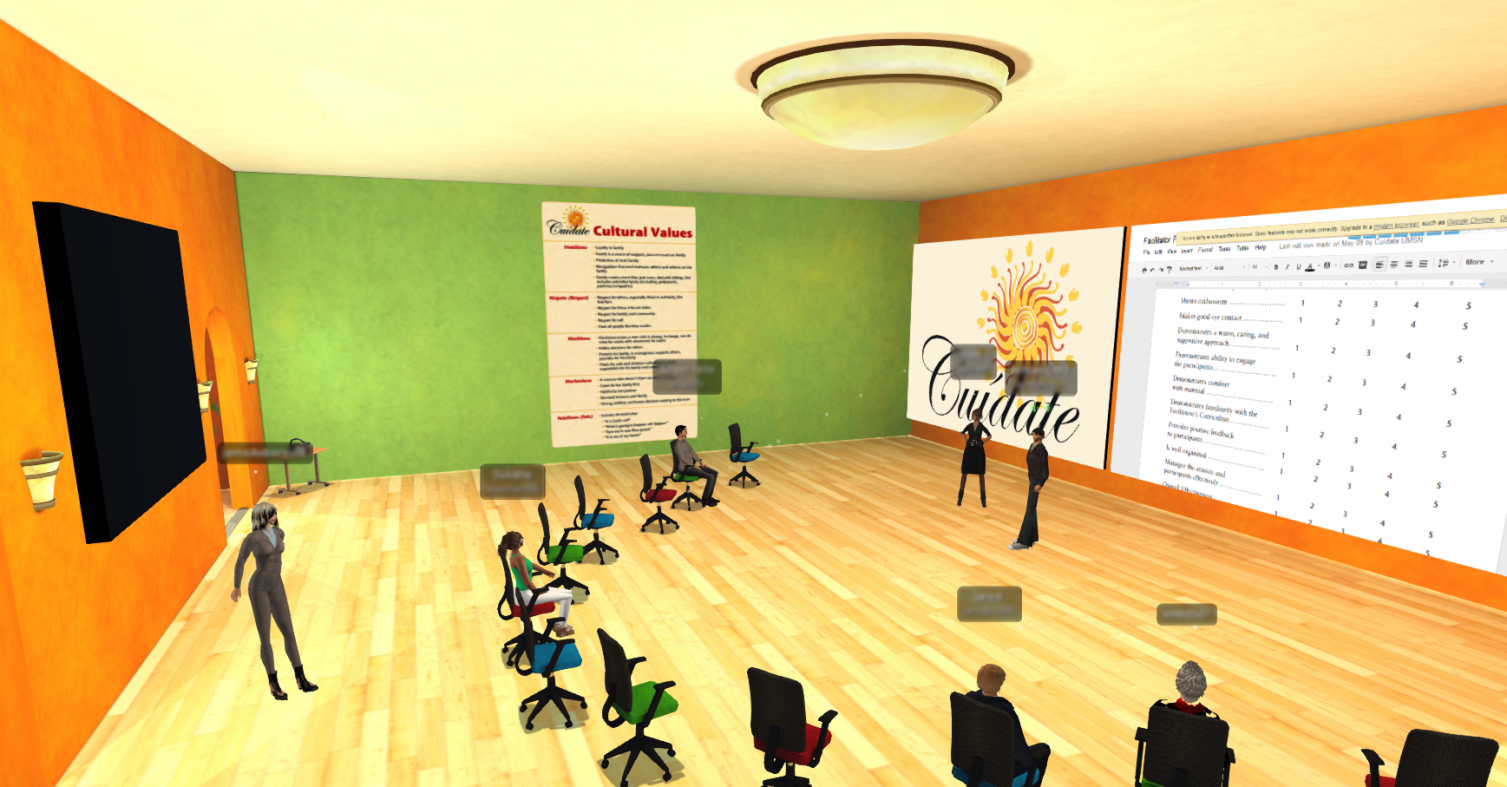
¡Cuídate! training room in Second Life.

A total of five *¡Cuídate!* training sessions were conducted in the SL environment (see Figure 1) with 35 facilitators (ie, participants) representing 24 agencies across the United States. These facilitators were recruited via networking and social media from CBOs across the country. Each training session group met three times. The first session was a 2.5-hour overview of the *¡Cuídate!* curriculum and overview of key SL features, followed 1 week later by two more sessions—4 hours and 3 hours in length, respectively—of *¡Cuídate!* content*.*

Facilitators ranged in age from 20 to 59 years, with the majority being female (24/35, 69%) and nearly half of Hispanic/Latino ethnicity (17/35, 49%). Education levels varied widely among the facilitators with a large number (28/35, 80%) having earned a bachelor’s degree or higher.

Comparisons were made with data from facilitators who participated in face-to-face training sessions (4 cohorts, 55 facilitators) held in St Louis, Philadelphia, Atlanta, and Memphis. These training sessions were held prior to the implementation of the SL *¡Cuídate!* training program. A total of 55 facilitators (41/55, 75% female) were trained in the face-to-face settings in Atlanta (19/55, 35%), St Louis (16/55, 29%), Philadelphia (12/55, 22%), and Memphis (8/55, 15%). Of the 55 trained facilitators, 44 (80%) surveys were collected from St Louis (14/16, 88%), Philadelphia (10/12, 83%), Atlanta (13/19, 68%), and Memphis (7/8, 88%) (personal communication, Danya International, Inc, 2014).

### Surveys

Facilitators in both the face-to-face and virtual training sessions were asked to complete pre- and posttraining surveys. These surveys included items measuring attitudes toward implementing *¡Cuídate!*, self-efficacy in working with Latino youth and in implementing *¡Cuídate!,* and overall evaluation of the *¡Cuídate!* training program. In addition, facilitators who participated in SL training were also asked to complete a modified version of the CoI survey. These items are described below.

### Evaluation of the Virtual Learning Environment

The community of inquiry framework survey instrument is a 34-item questionnaire measuring the three areas of cognitive presence, social presence, and teaching presence [10]. Original subscale alphas were found to be .94 (teaching presence), .91 (social presence), and .95 (cognitive presence). Further validity and reliability of the original instrument has been demonstrated through other studies [7,24].

Because the original instrument was designed for use in online courses, the CoI survey was adapted and reduced to 12 statements to measure the items that related to the *¡Cuídate!* Training of Facilitators*.* The items were scored on a 5-point Likert scale, ranging from 1 (strongly disagree) to 5 (strongly agree). Questions were retained in each of the three subscales. The first subscale—social presence—included six questions which measure the ability of facilitators to feel connected to each other in the absence of face-to-face contact. The teaching presence subscale was comprised of two questions that refer to ratings of the design, facilitation, and direction of processes needed to support learning. Finally, four questions measured cognitive presence which determine a learner’s ability to construct meaning through reflection. The modified CoI survey was administered only to the facilitators of the SL training. Cronbach alphas were high for each of the subscales: social presence (alpha=.89), teaching presence (alpha=.84), and cognitive presence (alpha=.85). Table 1 lists each of the statements from the survey.

Two questions were also asked to evaluate the overall experience in SL: (1) SL experience was an effective learning activity and (2) SL experience was a positive experience. These questions were rated on a 5-point Likert scale, ranging from 1 (strongly disagree) to 5 (strongly agree). Qualitative data were collected through a series of open-ended questions, several of which focused on aspects of the training in SL, as part of a debriefing survey. Specifically, facilitators were asked what they liked/disliked about the SL *¡Cuídate!* training program, what were the advantages/disadvantages to training in SL versus face to face, and how likely they were to participate in another or similar training program in SL.

To compare the face-to-face and SL training sessions, questions posed to facilitators in SL training sessions matched questions posed to facilitators trained in face-to-face sessions. These questions included evaluation of the training (eg, length and pace) and self-assessment of knowledge and skills (eg, knowledge of core elements and six modules, ability to respond to sensitive questions, and ability to help facilitators recognize how Latino culture supports safe sex). Items were scored on a 5-point Likert scale ranging from 1 (not confident) to 5 (very confident). Two additional questions were asked of SL facilitators to determine mastery of the curriculum and skills necessary to deliver the program (see Table 2 in the Results section)

All surveys for the SL facilitator training sessions (5 cohorts) were conducted using Qualtrics (Qualtrics, LLC). Links to each of the surveys were sent to facilitators via an email from the research team following completion of the training. Email reminders were sent to ensure a high response rate (35/35, 100%) and timely completion. Original surveys completed by the facilitators at the face-to-face sessions were scanned and sent via email to the research team.

**Table 1:** Community of inquiry survey statements (n=35).

Variables and statements		Second Life training score^a^, mean (SD)	Total subscale score, mean (SD)
**Social presence**			23.11 (4.12)
	I am comfortable conversing through an online medium.	3.77 (1.03)	
	I felt comfortable participating in training discussions.	4.17 (0.66)	
	I felt comfortable disagreeing with other facilitators while still maintaining a sense of trust.	4.06 (0.64)	
	Getting to know other facilitators gave me a sense of belonging.	3.86 (0.81)	
	Online or Web-based communication is an excellent medium for social interaction.	3.34 (1.08)	
	I was able to form distinct impressions of some course participants.	3.91 (0.92)	
**Teaching presence**			8.74 (1.01)
	The trainer helped keep facilitators on task.	4.31 (0.58)	
	The trainer provided feedback in a timely fashion.	4.43 (0.50)	
**Cognitive presence**			16.69 (1.97)
	I can describe ways to apply the knowledge I learned in training.	4.23 (0.55)	
	I was motivated to explore content-related questions.	4.14 (0.60)	
	Learning activities helped me construct explanations/solutions.	4.11 (0.58)	
	Reflection helped me understand fundamental concerns in training.	4.20 (0.63)	

^a^Scores are on a scale from 1 to 5, with 1 being strongly disagree and 5 being strongly agree.

### Analysis

To evaluate the facilitators' experience in the MUVE and examine if a learning environment was acceptable and supported the acquisition of learning outcomes, the CoI survey responses and qualitative responses in each of the three constructs—social presence, cognitive presence, and teaching presence—were analyzed together. To analyze the modified CoI survey and overall SL questions, descriptive statistics and frequency distributions were analyzed using SPSS version 21 (IBM Corp). A content analysis approach was used to analyze the nine open-ended questions with NVivo10 (QSR International) to code and organize the qualitative participant responses from the SL surveys.

For comparisons of face-to-face surveys and SL training sessions, responses from the four face-to-face sites (ie, St Louis, Philadelphia, Atlanta, and Memphis) were combined because there were no significant differences among training sites on any of the outcome variables (*P*>.05). Univariate frequencies of outcome variables were run and the Mann-Whitney U test for nonparametric data was used to make bivariate comparisons of the face-to-face and SL outcomes, as the data did not follow any specific parameterized distribution.

## Results

### Evaluation of the Virtual Learning Environment

#### Social Presence

Results indicate that most respondents experienced a moderately high level of social presence—in other words, they were able to feel connections with other participants. The subscale mean was 23.11 (SD 4.12) out of a possible 30. The highest ranking questions were “I felt comfortable participating in training discussions” (mean 4.17, SD 0.66), “I felt comfortable disagreeing with other facilitators while still maintaining a sense of trust" (mean 4.06, SD 0.64), and “I was able to form distinct impressions of some course facilitators” (mean 3.91, SD 0.92). Qualitative comments supported the survey findings. When asked what they most liked about SL, participants/facilitators noted particular aspects of the training that they liked best: “...very interactive and fun to see” and “...very interactive, very engaging.” Additionally, several comments reflected social connections—an indication of social presence—with others in the training session as an aspect of what they liked about SL training: “...interacting with people across the country” and “...interacting with other facilitators and getting feedback.”

Despite the positive comments, facilitators indicated that the lack of being able to see facial expressions was a disadvantage. For example, “The element of watching individual’s body language & facial expression is priceless...only thing MISSING” and “You can’t see everyone and their body language which is important when facilitating training but it [SL training] was very good.”

#### Teaching Presence

In general, facilitators reported a high perception of teaching presence. The subscale mean was 8.74 (SD 1.01) out of a possible 10. High mean scores were reported for items related to the trainers’ skill in keeping facilitators on task and also trainers’ ability to provide feedback in a timely fashion (mean 4.31, SD 0.58 and mean 4.43, SD 0.50, respectively). Qualitative comments supported a strong teaching presence: “A good balance between lecture and interactive activities was provided”—indicates design and facilitation—and “...getting feedback on our teach backs”—indicates facilitation. These comments indicated that the facilitator constructed activities in a way that supported learning in the environment.

#### Cognitive Presence

Cognitive presence was also highly rated. Cognitive presence supports the ability of learners to construct meaning through reflection and discourse. Overall, the subscale mean was 16.69 (SD 1.97) out of a possible 20. The majority of respondents were in agreement with statements indicating that they felt able to describe ways to apply knowledge learned in training (mean 4.23, SD 0.55), they were motivated to explore content-related questions (mean 4.14, SD 0.60), learning activities helped them construct explanations and solutions (mean 4.11, SD 0.58), and reflection helped them understand fundamental concerns in training (mean 4.20, SD 0.63). Qualitative comments that supported the survey findings were as follows:

Good to get better understanding from what is expected from the curriculum, sometimes you don’t know what the developer was thinking.

Understands the objectives of the course

Reinforcement of cultural values. Activities that reinforced the materials throughout

Understanding of the importance of how the cultural values are threaded throughout the curriculum

I definitely learned a lot—facilitation skills—appreciated knowledge everyone else brought

Met the objective of learning skills

The responses on the overall SL questions found that 69% of the participants (24/35) agreed/strongly agreed that SL was an effective learning activity and 77% of the participants (27/35) agreed/strongly agreed that SL was a positive experience.

### Acceptability of Second Life Training

The survey responses from the face-to-face and SL training sessions are presented in Table 2; bivariate comparisons between the face-to-face and SL training sessions are presented in Table 3.

A total of 80% of the participants (28/35) responded that the length of the training in SL was “about right” and 83% (29/35) responded that the pace of the training was “about right.” When asked about their self-assessment of knowledge and skills in every category—core elements, six modules, challenging questions, and recognize how Latino culture supports safer sex—over 50% of the participants in the SL training program reported, at a minimum, being confident with those skills.

**Table 2:** Comparison between Second Life training and CDC^a^ face-to-face training.

Variables and survey responses			CDC survey (n=44), n (%)	Second Life survey (n=35), n (%)
**Evaluation of training**				
	**Appropriateness of training length**			
		Too long	1 (2)	1 (3)
		A little too long	6 (14)	2 (6)
		About right	29 (66)	28 (80)
		A little too short	8 (18)	3 (8)
		Much too short	0 (0)	1 (3)
	**Pace of the training**			
		Much too slow	0 (0)	0 (0)
		A little slow	4 (9)	3 (8)
		About right	28 (65)	29 (83)
		A little fast	9 (21)	2 (6)
		Much too fast	2 (5)	1 (3)
**Self-assessment of knowledge and skills**				
	**Knowledge of core elements**			
		Not confident	0 (0)	0 (0)
		Little confident	0 (0)	0 (0)
		Somewhat confident	2 (5)	11 (32)
		Confident	16 (37)	19 (54)
		Very confident	25 (58)	5 (14)
	**Knowledge of six modules**			
		Not confident	0 (0)	0 (0)
		Little confident	0 (0)	0 (0)
		Somewhat confident	1 (2)	6 (17)
		Confident	21 (49)	24 (69)
		Very confident	21 (49)	5 (14)
	**Respond to challenging or sensitive questions/situations**			
		Not confident	0 (0)	0 (0)
		Little confident	0 (0)	0 (0)
		Somewhat confident	3 (7)	3 (8)
		Confident	17 (40)	16 (46)
		Very confident	23 (53)	16 (46)
	**Help participants recognize how Latino culture supports safer sex**			
		Not confident	0 (0)	0 (0)
		Little confident	0 (0)	0 (0)
		Somewhat confident	3 (7)	3 (9)
		Confident	16 (38)	17 (48)
		Very confident	23 (55)	15 (43)
**Mastery of *¡Cuídate!* curriculum**				
	**I have mastered content of program as written in manual**			
		Strongly disagree	N/A^b^	0 (0)
		Disagree	N/A	2 (6)
		In the middle	N/A	14 (40)
		Agree	N/A	18 (51)
		Strongly agree	N/A	1 (3)
	**I have mastered skills to deliver program as written in manual**			
		Strongly disagree	N/A	0 (0)
		Disagree	N/A	1 (3)
		In the middle	N/A	8 (23)
		Agree	N/A	20 (57)
		Strongly agree	N/A	6 (17)

^a^Centers for Disease Control and Prevention (CDC).

^b^Not applicable (N/A).

**Table 3:** Nonparametric comparisons of participant ratings between CDC^a^ face-to-face training and Second Life training.

Variable	CDC training score (n=44), mean (SD)	Second Life training score (n=35), mean (SD)	Mann-Whitney U test	*Z* score	*P* ^b^
Appropriateness of training length	3.00 (0.62)	3.03 (0.65)	765.50	-0.06	.96
Pace of the training	3.21 (0.68)	3.03 (0.51)	643.00	-1.41	.16
Knowledge of core elements	4.53 (0.59)	3.83 (0.66)	353.50	-4.35	<.001
Knowledge of six modules	4.47 (0.55)	3.97 (0.57)	441.50	-3.56	<.001
Respond to challenging or sensitive questions/situations	4.47 (0.63)	4.37 (0.65)	692.50	-0.67	.50
Help participants recognize how Latino culture supports safer sex	4.48 (0.63)	4.34 (0.64)	649.00	-0.98	.33

^a^Centers for Disease Control and Prevention (CDC).

^b^ *P* values (significance) based on two-tailed analysis.

### Comparison of Face-to-Face and Virtual Training Experience

There were no significant differences between participant ratings of the length or pace of training in SL as compared to face-to-face training (*P*=.96 and *P*=.16, respectively). Further, results indicate no significant difference between SL and face-to-face participant self-assessment of their ability to respond appropriately to sensitive questions and to recognize how Latino culture supports safer sex decisions (*P*=.50 and *P*=.32, respectively). There was a significant difference between reported knowledge of the components of the six *¡Cuídate!* modules (*P*<.001). Those facilitators who participated in face-to-face training sessions had higher knowledge scores (mean 4.44, SD 0.56) as compared to those who participated in SL training sessions (mean 3.97, SD 0.57).

Additionally, there was a significant difference (*P*<.001) between SL and face-to-face participant ratings of their ability to describe the six core elements of the *¡Cuídate!* curriculum. Facilitators who participated in the face-to-face training sessions rated their level of confidence in their knowledge of the core elements and six modules significantly higher (mean 4.47, SD 0.62) compared to those in the SL training sessions (mean 3.83, SD 0.67). However, of the SL participants, 54% (19/35) agreed/strongly agreed that they mastered the content of the program as written in the manual, and 74% (26/35) agreed/strongly agree that they mastered the skills to deliver the program as written in the manual.

## Discussion

### Principal Findings

MUVEs hold promise for delivering training in the future without the training-related travel costs, thereby increasing the use of these crucial programs that have widespread and critical influence on population health and health outcomes. It is always challenging to deliver training in a Web-based environment when you cannot physically see the facilitators, particularly when training requires active participation rather than passive receipt of knowledge. Part of the *¡Cuídate!* Training of Facilitators curriculum [21] requires facilitators to conduct teach backs, necessitating engagement with others. Consequently, the use of a MUVE such as SL over a noninteractive format (eg, webinar) is essential to support the interaction necessary to support the role-play interaction. Results of this study indicate that SL is an acceptable and feasible way to deliver training and achieve outcomes that lead to learning success.

The results of this study indicate that the three constructs of social, teaching, and cognitive presence were present in the SL environment. The facilitators also rated the SL experience as positive and effective. Specifically, the facilitators experienced a moderately high level of social presence in the SL environment. This connectedness to others has been shown to be a predictor of success in online courses [11]. Although virtual environments will never duplicate the social presence found in face-to-face environments, the comments demonstrated how engaged and immersed in the environment the facilitators were when responding (eg, “interacting with people across the country"). The facilitators only "met" or interacted in SL, yet they felt a connection to others with whom they trained. This is consistent with what was seen in other studies using SL [14-16].

In relation to cognitive presence—an indicator of how well our facilitators achieved the learning objectives—facilitators agreed the activities were helpful and they felt capable of applying the knowledge gained in training. Facilitators had access to all six modules and all the activities in the training manual; they were asked to review all modules/activities as part of their prelearning work prior to coming to training. Also noted in the training survey, the majority of the participants agreed they had mastered the skills to deliver the curriculum, and over half agreed they had mastered the content of the program. This is consistent with the study by Schwaab [16] in which MI skills improved after training in SL. Teaching presence was also supported by the SL environment. Facilitators rated the trainers high in keeping them on task and providing them with timely feedback, while comments supported a good balance of activities. Comparison studies did not rate the quality of teaching presence.

There were no differences between face-to-face- and SL-trained facilitators in their confidence levels regarding their ability to respond to challenging or sensitive questions when delivering the curriculum, which is important to the mastery of the curriculum. This is essential to meeting the aim of ensuring facilitators have the ability to deliver the curriculum effectively. The significant difference between participants' self-assessment of their abilities to identify the core elements and modules was not surprising. During the face-to-face training sessions, facilitators were given an overview of the six modules, whereas facilitators in the virtual environment were required to review all materials ahead of time as part of the prelearning work. Given the complexities and multiple priorities of the facilitators, they may have not reviewed the materials or did not thoroughly review them prior to the virtual training. Further work is needed to ensure facilitators complete all prelearning materials. This can be done by requiring interaction with the modules as they are being presented or with a postmodule quiz.

The high ratings on the mastery indicate that although participants did not experience all learning in SL, they did feel they had the skills to deliver the content. This finding supports the efficacy of conducting training in SL. Educators should consider this when trying to minimize time spent in training while ensuring mastery of the content and various learning methodologies. Eliminating extra time in training will assist with efficient use of the limited resources available to community-based organizations or other groups pursuing training.

### Conclusions

As it becomes more challenging to access training to deliver EBIs, alternative methods like training in virtual worlds need to provide access to training in a manner that is both effective and acceptable to those receiving the training. This will then open a way to increase access to even remote areas, provided there is Internet access and a willingness to engage in a virtual training environment. This study demonstrates that training can be effectively delivered in a virtual world and the training environment in SL can be designed and delivered in a manner that is acceptable to the participants. SL was an effective training environment for the facilitators to achieve the ability to learn the skills needed to deliver the *¡Cuídate!* curriculum, including demonstration of the teach backs that are essential to being able to effectively deliver this successfully to Latino youths.

## Abbreviations

CBO: community-based organization
CDC: Centers for Disease Control and Prevention
CoI: community of inquiry
EBI: evidence-based intervention
MBA: Master of Business Administration
MI: motivational interviewing
MUVE: multi-user virtual environment
N/A: not applicable
SL: Second Life
STD: sexually transmitted disease
